# Reaching adolescents with health services in Nepal

**DOI:** 10.2471/BLT.17.020217

**Published:** 2017-02-01

## Abstract

Sexual and reproductive health services for adolescents are being rolled out in Nepal, but many young people have yet to benefit. Shiva Raj Mishra reports.

Radha (not her real name), a 15-year-old resident of Kathmandu, the capital of Nepal, read about sexual and reproductive health in her school textbook, but it did not answer all of her questions.

When she asked her parents, they did not want to discuss this traditionally taboo topic.

Radha’s story is typical, says Amit Timilsina, who heads nongovernmental organization YUWA (“youth” in Nepali) that is helping to provide young people like Radha with sex education and reproductive health information. 

“Adolescents are shy about asking questions and learning about sexual and reproductive health in the classroom, and they don’t always receive support at home to seek out the information and services they need,” Timilsina says.

About 22% (6.38 million) of Nepal’s 28.5 million population (government projection for 2016) are adolescents aged 10–19 years. The legal age of marriage in Nepal is 20 years. Despite that, 48.5% of women aged 20–49 years were married by the age of 18 and 15.5% aged 15-49 were married by the age of 15, according to the *Nepal Multiple Indicator Cluster Survey 2014 (NMICS 2014)*.

Childbearing also begins early, especially in rural areas. Almost a quarter of women in Nepal give birth before the age of 18 and nearly half before they are 20 years old, according to the *Nepal demographic and health survey 2011 (NDHS 2011)* although the numbers of teenage pregnancies have reduced in recent years, as shown by *NMICS* 2014.

Early childbearing can have negative health consequences for teenage mothers and their infants. Moreover, it greatly reduces girls’ education and employment opportunities.

High rates of adolescent marriage and the pressure to bear a child – preferably a son – right after marriage are often cited as key factors holding back economic development, helping to make Nepal one of the poorest countries in the world.

In a bid to better meet the health needs of adolescents, the Nepali government launched a national programme in 2010 to provide adolescent-friendly sexual and reproductive health services as part of its five-year health sector plans.

The National Adolescent Sexual and Reproductive Health Programme aims to serve all adolescents. Although launched in the era of the Millennium Development Goals that ended in 2015, the programme is in the spirit of the sustainable development goals (2015–30) that stress universal access to health care and leaving no one out.

The programme is complemented by sex education. Between 2002 and 2006, with the support of the United Nations Population Fund (UNFPA), Nepal introduced what is known as comprehensive sexuality education in schools as part of the national curriculum. 

The approach stresses human rights and gender equality, and helps adolescents develop the life skills they need to cope with puberty and weigh the risks of early marriage and pregnancy.

“We are working with the Ministry of Education to empower teachers and students with training and information on this important topic,” says Manju Karmacharya, the Adolescent Sexual and Reproductive Health Programme Officer with UNFPA in Kathmandu. 

NGOs also play a role. YUWA has been complementing this school-based intervention with peer education in 14 districts. 

“Young people may feel inhibited discussing these issues in the classroom, so other ways of delivering sexuality education are needed,” Timilsina says, adding that his NGO plans to provide such support via mobile phone applications from April.

Since the National Adolescent Sexual and Reproductive Health Programme was launched, services – including counselling, provision of contraceptives and screening for sexually transmitted infections – have been rolled out at 1134 health facilities in 63 of Nepal’s 75 districts.

The remaining districts will be covered by 2021, according to Ghanashyam Pokharel, former chief of Adolescent Sexual and Reproductive Health and Family Planning from the Family Health Division in the health ministry. 

“The idea is to create an environment that is conducive for adolescents to visit health facilities and receive health services that address their specific concerns,” Pokharel says.

To ensure that these centres are adolescent friendly and that services are of high quality, the Family Health Division developed a Quality Improvement and Certification Tool in 2015 with UNFPA support. To date, 29 centres have been certified through a rigorous process that includes focus group discussions with users. 

“We are supporting the provision of quality services to adolescents by training health service providers and equipping heath facilities, so that they can be certified as truly adolescent friendly,” Karmacharya explains.

Given the long time frame for implementation, the programme’s impact on reducing early marriage and pregnancy and improving adolescent health has not yet been evaluated.

A mid-term review of the programme in 2013 and a study in 2014 found several barriers to its implementation, including poor ownership at local level and poor integration with other health programmes.

A new adolescent and health development strategy seeking to address these and other shortcomings was recently completed, with support from Save the children, WHO and other partners, Pokharel says.

“The new strategy takes a more holistic approach by integrating adolescent sexual and reproductive health services better with other health services.”

Ghanashyam Pokharel

“The new strategy takes a more holistic approach by integrating adolescent sexual and reproductive health services better with other health services,” Pokharel says.

“It covers prevention of noncommunicable diseases, such as education on the harms of smoking tobacco, and it covers mental health including suicide prevention, as suicide is the leading cause of death among adolescents in Nepal,” Pokharel says.

Pokharel notes other positive developments: “Health workers have now started to believe that adolescents are entitled to understand adolescent sexual reproductive health issues and to receive these services”.

But for many vulnerable young people, these services remain inaccessible. Some adolescents still do not know where the nearest services are located, while some do not know they even exist. In mountainous areas, these services are difficult to reach because of poor roads and inadequate public transport.

“The cost of food and lodging while travelling to health centres and out-of-pocket spending on medicines are also barriers to the utilization of these services,” says Pushkar Raj Silwal, a technical adviser from the Deutsche Gesellschaft für Internationale Zusammenarbeit (GIZ), a German government enterprise that supports the programme.

“In Nepal, we have seen tremendous progress in maternal and child health in past decades, but this has not been reflected in adolescent health,” Silwal says.

“We hope to make these sexual and reproductive health services more accessible.”

Manju Karmacharya

UNFPA has also been supporting Nepal in the planning and implementation of the programme. “We hope to make these sexual and reproductive health services more accessible by providing adolescent-friendly information corners and comprehensive sexuality education in schools, and by providing social and financial skills guidance to school leavers,” says Kamarcharya.

For Shristi Rijal, another activist with YUWA, adolescents need evidence-based information to counter misconceptions and local superstitions. She cites the tradition of *chhaupadi* that regards women as impure during their monthly period.

“Women and girls are banished to the cowshed until sunrise on the fifth day, exposing them to rape, attacks by wild animals and the cold,” Rijal says, adding that the practice has been banned for more than a decade, in vain.

Last December, the story of a 15-year-old Nepali girl, who suffocated to death in the family cowshed after lighting a fire to keep warm, was widely reported in national and international media.

The massive earthquakes that wrought death and destruction across much of Nepal in 2015 also brought challenges for the Adolescent Sexual and Reproductive Health Programme. About 500 health facilities in 14 districts were damaged, depriving an estimated 1.2 million adolescents of these services.

Another NGO, Visible Impact, helps to equip young women and girls in the earthquake-affected areas with skills and knowledge to improve their sexual and reproductive health.

“In the past, health workers were reluctant to give adolescents contraceptives as they feared they would initiate sex too early,” says Medha Sharma, who heads Visible Impact. “Now giving them condoms is routine.”

The unmet need for contraception in Nepal remains high. About 47% of adolescents aged 15 to 19 years who need contraception, do not have it, according to *NMICS* 2014.

For Karmacharya, the key challenges for the National Adolescent Sexual and Reproductive Health Programme are in integrating services for adolescents into existing sexual and reproductive health services, quality assurance and funding.

“Funding is far from being adequate to offer comprehensive adolescent-friendly health services covering all aspects of adolescent sexual and reproductive health and comprehensive sexuality education to all districts,” she says.

**Figure:**
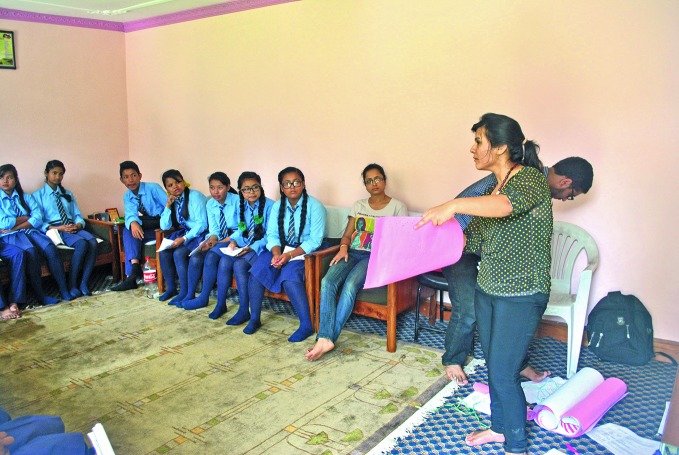
A school-based workshop on menstrual health given by nongovernmental organization, Visible Impact.

Courtesy of Visible Impact

**Figure:**
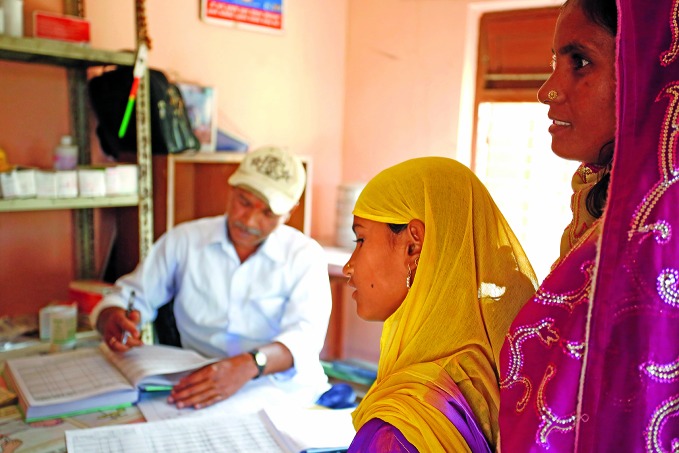
A 10-year-old girl talks to a health worker at Bharatpur Health Post in Mahottari district as her aunt looks on.

© UNFPA Nepal

